# Supplementary material for: Lessons from the deployment and management of public handwashing stations in response to the COVID-19 pandemic in Kenya: A cross-sectional, observational study
Source: PLoS One. 2024 Jun 6;19(6):e0303073. doi: 10.1371/journal.pone.0303073 (PMC11156298; doi:10.1371/journal.pone.0303073)
Supplement: S1 Equation — (DOCX) [file pone.0303073.s001.docx]

# Supplementary Information

**Supplementary Equation 1: Sampling Calculation for Determining Study Size**

$$n=\left\{ \frac{\frac{\left( Z \right)^{2}\times P\left( 1-P \right)}{\left( e \right)^{2}}}{1+\frac{\left( Z \right)^{2}\times P\left( 1-P \right)}{\left( e \right)^{2}N}}*deff \right\}$$

Where

- **Z** = Selected critical value of the desired level of confidence. We use a 95% confidence level that corresponds with Z=1.96
- **P** = prevalence (in this study, it is assumed that there is a 50-50 chance of someone visiting a HWS) = 0.5
- **e** = acceptable margin of error for this study= 5%
- **N** = population=deployed HWS=5,293
- **deff** = design effect; due to the effect of stratification, the study will assume a design effect of 1.5

Reference

1. Organization WH. Hand Hygiene: Why, How & When. 2009.
