# Supplementary material for: Lessons from the deployment and management of public handwashing stations in response to the COVID-19 pandemic in Kenya: A cross-sectional, observational study
Source: PLoS One. 2024 Jun 6;19(6):e0303073. doi: 10.1371/journal.pone.0303073 (PMC11156298; doi:10.1371/journal.pone.0303073)
Supplement: S1 Table — (DOCX) [file pone.0303073.s002.docx]

**S1 Table:**

| **County** | **Partner** | **Paid caretakers** | **Unpaid caretakers** | **Deployment phase** | **Volume of HWS** | **Total** |
| --- | --- | --- | --- | --- | --- | --- |
| Nairobi | SHOFCO | 86 | 0 | Phase III | 100 L | 86 |
|  | JJK | 0 | 30 | Phase III | 100 L | 30 |
|  | Rotary | 14 | 50 | Phase I & II | 20 L & 60 L | 64 |
|  | Sanergy | 0 | 15 | Phase III | 100 L | 15 |
| Kwale | Shujaaz | 0 | 55 | Phase II | 60 L | 55 |
| Embu | Copia | 0 | 70 | Phase I & II | 20 L & 60 L | 70 |
| Mombasa | BRCK | 0 | 60 | Phase III | 100 L | 60 |
|  | Rotary | 0 | 20 | Phase II & III | 60 L & 100 L | 20 |
| Homabay | Copia | 0 | 10 | Phase I | 20 L | 10 |
|  | Rotary | 0 | 20 | Phase I | 20 L | 20 |
| **Total** |  | **100** | **330** |  |  | **430** |
| L, litres; HWS, handwashing station; SHOFCO, Shining Hope for Communities | | | | | | |
