# Supplementary material for: Lessons from the deployment and management of public handwashing stations in response to the COVID-19 pandemic in Kenya: A cross-sectional, observational study
Source: PLoS One. 2024 Jun 6;19(6):e0303073. doi: 10.1371/journal.pone.0303073 (PMC11156298; doi:10.1371/journal.pone.0303073)
Supplement: S2 Table — (DOCX) [file pone.0303073.s003.docx]

**S2_Table**

|  | **N=316** | **%** |
| --- | --- | --- |
| **Presence/placement** |  |  |
| Shopping centre | 173 | 54.7 |
| Markets | 34 | 10.8 |
| Bus terminus | 19 | 6 |
| Government facilities | 16 | 5.1 |
| Roadside | 13 | 4.1 |
| Public toilets | 9 | 2.8 |
| Places of worship | 8 | 2.5 |
| Schools | 8 | 2.5 |
| Community Hall | 5 | 1.6 |
| Health Facility | 4 | 1.3 |
| Other | 27 | 8.5 |
| **Visibility** | | |
| Easily visible | 193 | 61.1 |
| Visible if known location | 117 | 37.0 |
| Difficult to find | 6 | 1.9 |
| **Accessibility of the HWS*** | | |
| **HWS accessible for a person using a wheelchair** | 111 | 35.1 |
|  |  |  |
| **HWS accessible to children under 12yrs** | 243 | 76.9 |
|  |  |  |
| **HWS accessible to older persons** | 241 | 76.3 |
|  |  |  |
| **IEC materials about handwashing visible on or near the tank** | 148 | 46.8 |
| **IEC Material glued on a tank**** | | |
| It is at eye level and easy to read while using the HWS | 142 | 95.9 |
| It is on the tank but not at eye level whilst using the HWS | 6 | 4.1 |

* Accessibility was defined as HWS that are easily visible (spotted), conveniently reached by users and had visible nudges/IEC materials. HWS accessibility for children under 12 years and older persons was determined based on ease of access and suitability for use by these age groups. This designation does not imply a height requirement but rather encompasses features such as the height and accessibility of the handwashing station components (e.g., taps, soap dispensers) that facilitate ease of use for children under 12 years and older persons.

** Only accessible to users immediately near the facility as other objects, such as market stalls, make the facility less visible.
