# Supplementary material for: Lessons from the deployment and management of public handwashing stations in response to the COVID-19 pandemic in Kenya: A cross-sectional, observational study
Source: PLoS One. 2024 Jun 6;19(6):e0303073. doi: 10.1371/journal.pone.0303073 (PMC11156298; doi:10.1371/journal.pone.0303073)
Supplement: S3 Table — (DOCX) [file pone.0303073.s004.docx]

**S3_Table**

| **Washed hands using proper technique*** | **IEC Materials available on the handwashing stations** | | **Total n (%)** |
| --- | --- | --- | --- |
|  | **No n (%)** | **Yes n (%)** |  |
| Yes | 79 (41.6) | 114 (54.5) | 193 (48.4) |
| No | 111 (58.4) | 95 (45.5) | 206 (51.6) |
| **Total n (%)** | 190 (100) | 209 (52.4) | 399 (100) |

* Proper technique as defined by WHO (hands washed with soap for at least 40 seconds)(1).

Chi-square test: X² = 6.70, p = 0.0096
