# Supplementary material for: Lessons from the deployment and management of public handwashing stations in response to the COVID-19 pandemic in Kenya: A cross-sectional, observational study
Source: PLoS One. 2024 Jun 6;19(6):e0303073. doi: 10.1371/journal.pone.0303073 (PMC11156298; doi:10.1371/journal.pone.0303073)
Supplement: S4 Table — (DOCX) [file pone.0303073.s005.docx]

**S4_Table**

|  | **N** | **%** |
| --- | --- | --- |
| **Location of soap, n=316** |  |  |
| Next to the water tank | 247 | 78.2 |
| On top of the water tank | 29 | 9.2 |
| In the custody of the facility manager | 5 | 1.6 |
| Soap not found | 35 | 11.1 |
|  |  |  |
| **Soap status, n=81** |  |  |
| Soap looks dirty | 41 | 13.0 |
| Soap looks clean | 40 | 12.7 |
|  |  |  |
| **Soap brand status, n=316** |  |  |
| Visibly branded | 54 | 17.1 |
| Tale characteristics of branding | 24 | 7.6 |
| No tale characteristics of branding | 203 | 64.2 |
| Missing | 35 | 11.1 |
|  |  |  |
| **Ease of tap use, n=316** |  |  |
| The tap opens and closes easily | 275 | 87.0 |
| Difficult to open or close but works | 33 | 10.4 |
| The tap does not work at all | 7 | 2.2 |
| There is a foot pedal operating the tap | 1 | 0.3 |
|  |  |  |
| **Height of handwashing station, n=316** |  |  |
| Must bend to use the handwashing station | 44 | 13.9 |
| The station is at a comfortable height | 258 | 81.6 |
| The handwashing station is too high | 14 | 4.4 |
|  |  |  |
| **Water drains** |  |  |
| Directly onto the ground | 64 | 20.3 |
| Wash basin, emptied by manager | 122 | 38.6 |
| Wash basin connected to another container | 65 | 20.6 |
| Wash basin connected to a pipe that drains onto the ground | 65 | 20.6 |
