# Supplementary material for: Lessons from the deployment and management of public handwashing stations in response to the COVID-19 pandemic in Kenya: A cross-sectional, observational study
Source: PLoS One. 2024 Jun 6;19(6):e0303073. doi: 10.1371/journal.pone.0303073 (PMC11156298; doi:10.1371/journal.pone.0303073)
Supplement: Attachment — Submitted filename: Response to Reviewers.docx [file pone.0303073.s006.docx]

Dear Editor,

Thank you for giving us the opportunity to submit a revised draft of our manuscript for publication in your esteemed journal. We appreciate the time and effort that you and the reviewers have dedicated to providing feedback on our manuscript and are grateful for the insightful comments on our paper. We have incorporated most of the suggestions made by the reviewers. Those changes are tracked within the updated manuscript. Please see below a point-by-point response to reviewers' comments and concerns. These revisions have improved the manuscript. On behalf of my co-authors, I thank you for your consideration of this revised manuscript.

Sincerely,

Dennis Munai (corresponding author)

**Academic Editor:**

1. **Please ensure that your manuscript meets PLOS ONE's style requirements, including those for file naming.**

**Response:**

*We appreciate the opportunity to address this matter and ensure that our manuscript adheres to PLOS ONE's style guidelines, including those for file naming. Upon review, we have carefully revised the manuscript files to meet the specified style requirements. We have meticulously formatted the document and ensured that all file names comply with the prescribed conventions outlined by PLOS ONE. We apologize for any oversight in our initial submission and appreciate your guidance in ensuring that our manuscript meets the necessary standards.*

2. **Please report in the Methods section the day, month and year of the start and end of the participant recruitment period for this study.**

**Response:**

*Thank you for your suggestion, the recruitment period for this study has now been included from lines 157 and 158 in the data collection methods.*

3. **We suggest you thoroughly copyedit your manuscript for language usage, spelling, and grammar. If you do not know anyone who can help you do this, you may wish to consider employing a professional scientific editing service.**

**Response:**

*Thank you for your suggestion. The manuscript has undergone copyedit. The contacts of the copy editor is as below:*

**Name:** Joyce Nyambura

**Cell:** +254721543021

**Email:** [joycelesham@gmail.com](mailto:joycelesham@gmail.com)

4. **In this instance it seems there may be acceptable restrictions in place that prevent the public sharing of your minimal data. However, in line with our goal of ensuring long-term data availability to all interested researchers, PLOS' Data Policy states that authors cannot be the sole named individuals responsible for ensuring data access (**[**http://journals.plos.org/plosone/s/data-availability#loc-acceptable-data-sharing-methods**](http://journals.plos.org/plosone/s/data-availability#loc-acceptable-data-sharing-methods)**).**

**Before we proceed with your manuscript, please also provide non-author contact information (phone/email/hyperlink) for a data access committee, ethics committee, or other institutional body to which data requests may be sent. If no institutional body is available to respond to requests for your minimal data, please consider if there any institutional representatives who did not collaborate in the study, and are not listed as authors on the manuscript, who would be able to hold the data and respond to external requests for data access? If so, please provide their contact information (i.e., email address). Please also provide details on how you will ensure persistent or long-term data storage and availability.**

**Response:**

*We appreciate the opportunity to fulfil this requirement to provide non-author information for a data access committee, ethics committee, or other institutional body to which data requests may be sent in relation to our manuscript. Please find below the contact information for the designated institutional body responsible for handling data access requests:*

- *Institution Name/Department: Amref Ethics and Scientific Review Committee*
- *Contact Person's Name: Mr. Samuel Muhula*
- *Contact Person's Position: Senior Manager, Learning and Impact*
- *Contact Person's Email:* [*Samuel.muhula@amref.org*](mailto:Samuel.muhula@amref.org)
- *Contact Person's Phone Number: +254 721958734*

*This contact information is provided to facilitate communication and access to the data used in our study. We assure you that the designated contact person is authorized to handle data access requests on behalf of our institution and will respond promptly to inquiries regarding data availability and access. Please feel free to contact us if you require any further information or clarification regarding the provided contact details.*

5. **We note that Figures 1 and 4 in your submission contain copyrighted images. All PLOS content is published under the Creative Commons Attribution License (CC BY 4.0), which means that the manuscript, images, and Supporting Information files will be freely available online, and any third party is permitted to access, download, copy, distribute, and use these materials in any way, even commercially, with proper attribution. For more information, see our copyright guidelines:** [**http://journals.plos.org/plosone/s/licenses-and-copyright**](http://journals.plos.org/plosone/s/licenses-and-copyright)**. We require you to either (1) present written permission from the copyright holder to publish these figures specifically under the CC BY 4.0 license, or (2) remove the figures from your submission.**

**Response:**

*We respectfully assert that the images included in Figures 1 and 4 are original creations produced by our research team and do not breach any copyrights. As per your guidelines, we assure you that our manuscript, including the images in question, adheres to this licensing agreement. These images have not been previously published or distributed elsewhere. We appreciate the thorough review and attention to detail by the editorial team. We are confident that the inclusion of Figures 1 and 4 contributes significantly to the scientific merit and integrity of our manuscript.*

6. **We note that Figure 2 in your submission contain [map/satellite] images which may be copyrighted.**

**Response:**

*We appreciate the meticulous review conducted by the editorial team and the opportunity to address the concerns regarding the inclusion of map/satellite images in Figure 2. We wish to clarify that the image presented in Figure 2 is an original artwork/drawing created by our research team. The drawing was produced in-house specifically for this research project and does not contain any copyrighted materials or third-party content.*

7. **Please reduce the words in the abstract to 250 words.**

**Response:**

*We have revised the abstract to meet the suggestion of reducing the word count. The revised abstract now contains fewer than 250 words while still effectively summarizing the key findings of the study.*

8. **I don't see the knowledge gap in the introduction.**

**Response:**

*We appreciate the reviewer's diligence in assessing the clarity of our manuscript. We've taken note of the comment regarding the identification of the knowledge gap and have since revised the Introduction section to provide a more detailed description of the gap our study seeks to address. Our study aims to bridge the existing research gap surrounding the functionality, usability, and maintenance of handwashing stations in public settings amid the COVID-19 pandemic. By conducting a comprehensive observational study, we aimed to shed light on the operational challenges and user perceptions associated with these facilities, particularly in the context of the pandemic response efforts. Through our findings, we endeavour to contribute valuable insights that can inform the optimization of hand hygiene practices and the implementation of effective public health interventions. We believe that these revisions adequately address the reviewer's concern and enhance the clarity of our manuscript. This modification is located in the last four paragraphs of the Introduction section.*

9. **I think lines 81-91 on page 4 should be put in the method, also the line…..**

**Response:**

*Thank you for your suggestion regarding the relocation of the paragraph from lines 81-91 on page 4 to the Methods section. We agree that this information is integral to understanding the methodology of our study. We have incorporated this paragraph into the Methods section under the subheading titled "Study setting and Population".*

10. **I suggest removing the sampling calculation to supplementary material**

**Response:**

*We appreciate your feedback regarding the inclusion of the sampling calculation in the main manuscript. Upon careful consideration, we agree that moving the sampling calculation to the supplementary material will improve the flow and readability of the main text. We have relocated the sampling calculation to the supplementary material, providing a clear reference in the main text to facilitate easy access for readers who wish to review the details. This adjustment will streamline the presentation of the study methods while ensuring that essential details remain accessible to interested readers.*

11. **Line 199-202 -> Why is the font size different?**

**Response:**

*Thank you for bringing attention to the discrepancy in font size. We have ensured that the font size is uniform throughout the entire document by standardizing it to the appropriate size as per the journal's guidelines.*

12. **Line 246-249 -> only one asterisk for two footnotes?**

**Response:**

*Thank you for bringing this to our attention. We appreciate your careful review of our manuscript. We have revised the Accessibility of Handwashing Stations section to address the issue of the footnotes. Each footnote now has its corresponding asterisk, as follows:*

*"* Accessibility was defined as HWS that are easily visible (spotted), conveniently reached by users and had visible nudges/IEC materials.*

*** Only accessible to users immediately near the facility as other objects, such as market stalls, make the facility less visible."*

13. **Line 259-260 -> It is strange that the quote appears here without any explanation/narration before.**

**Response:**

*We appreciate your valuable feedback regarding the incorporation of quotes within the manuscript. In response to your concern about the quote appearing without preceding explanation or narration, we have revised the section on "Accessibility of Handwashing Stations in Public Spaces" to seamlessly integrate the quote within the context of the discussion. As suggested, we have introduced the quote immediately after presenting the findings related to the accessibility of handwashing stations.*

14. **Table 3 -> Please report statistical results properly.**

**Response:**

*Thank you for your feedback. We have revised Table 3 to present the association between washing hands using the proper technique and the presence of information, education, and communication (IEC) materials on handwashing stations more clearly. In the updated table, we have split the merged cells regarding the availability of IEC materials, presenting them separately under "Yes" and "No" categories. The total column is now standalone. Furthermore, we have removed the information on the Chi-square test from the last row and placed it after the footnote, providing a cleaner presentation of the statistical results.*

15. **I think the 5 tables (and some of them are big tables) are too much. Please consider reducing them and putting some in the supplementary.**

**Response:**

*Thank you for your suggestion regarding the number of tables included in the manuscript. We have carefully considered your feedback and have made adjustments accordingly. Tables 1, 2, 3, and 4 have been relocated to the supplementary material, as suggested, to streamline the main manuscript and improve its readability.*

16. **There should be a conclusion section. Please make the last paragraph in the discussion as your conclusion section.**

**Response:**

*Thank you for your feedback. We have revised the manuscript accordingly. The last paragraph in the discussion section now serves as the conclusion section, encapsulating the key findings and implications of the study.*

**Reviewer 1**:

Thank you for the opportunity to review the manuscript titled: Lessons from the deployment and management of public handwashing stations in response to the COVID-19 pandemic in Kenya: A Cross-sectional, observational study.

The authors sought to assess the functionality, usability and accessibility of rolled public handwashing stations in a COVID-19 pandemic context to improve their operation and maintenance and to inform strategies for future rollouts. The authors referenced multiple complementary methodologies and primarily found that the distribution of public handwashing facilities can have a limited lifetime, highlighting some implications of their findings. On the whole the manuscript was an interesting read and of value to PLoS One. There are some opportunities to bolster the manuscript. The main feedback is that additional clarification of the methods/measures and alignment of the results per the listed methodologies is sorely needed.
Here are my detailed comments and suggestions:

1. **Abstract**
a) Line 21: clarify that financial support was also provided.

**Response:**

*Thank you for your valuable feedback. We have revised line 21 to now indicate that there were caretakers who were supporte financially to operate and manage the handwashing stations.*

b) Include other results- qualitative and exit surveys. Quantitative data were collected through spot checks, caretaker surveys, user observations and user-exit interviews; whereas qualitative data were collected using key informant interviews, focus group discussions and in-depth interviews.

**Response:**

*Thank you for your valuable feedback. We have revised the abstract to include additional results from the qualitative surveys. It now reads, 'Qualitative data highlighted challenges such as inadequate signage, limited availability of soap and water, and issues related to the physical accessibility of the stations, particularly for individuals with disabilities.'*

2. **Introduction**
a) 89: Include that fabricated stands, soap and soap holders were also a part of phase III.

**Response**:

*We appreciate your attention to detail and believe that this clarification strengthens the description of the intervention in phase 3. It now clearly states, 'In this final phase, 1558 brightly coloured 100 litre-capacity HWS with wastewater drainage pipes, fabricated stands, soap and soap holders, and IEC posters were distributed.' Please note that because of the editor's comments we moved this information to the Methods section under study setting and population. Please note that this paragraph has been moved to the Methods section under the 'Study setting and population' sub-heading.*

b) 98: define what it meant by (O&M) and the specific tasks the caretakers were to carry out. It is unclear how soap and water were provided.

**Response**:

*In response to this query, we acknowledge the need for clarification regarding the term (O&M), which stands for operation and maintenance. In the context of our study, operation and maintenance encompassed a range of responsibilities assigned to the handwashing station caretakers. These tasks included ensuring the availability of soap and water, regular cleaning of the handwashing stations, refilling water containers, and restocking soap as needed. As for the provision of soap and water, these essential supplies were typically provided by the caretakers themselves or by individuals in the community.*

*It now reads, 'Each handwashing station (HWS) was assigned a caretaker responsible for its operation and maintenance. In the context of our study, operation and maintenance encompassed a range of responsibilities assigned to the handwashing station caretakers. These tasks included ensuring the availability of soap and water, regular cleaning of the handwashing stations, refilling water containers, and restocking soap as needed. Regarding the provision of soap and water, guidelines were established during deployment, emphasizing the responsibility of HWS recipients to regularly refill water and replenish soap for handwashing once the initial supply of 800mg provided by coalition partners was depleted. This task was undertaken either by the caretakers or other community members. The caretakers fell into two categories: volunteers, typically local business owners, or paid individuals. Paid caretakers, predominantly stationed in informal settlements, were contracted by non-governmental organizations affiliated with the National Business Compact Coalition (NBCC) for periods ranging from 3 to 6 months, coinciding with the peak distribution of HWS and COVID-19 caseload. Their role was to remain on standby, positioned next to or in close proximity to the HWS, to ensure optimal operation and maintenance. On the other hand, unpaid caretakers were volunteers residing near the HWS, often near their businesses such as stalls, shops, supermarkets, markets, or transport hubs. These caretakers, presumed to be respected members of the community, were expected to assume responsibility for the operation and maintenance of the HWS without formal compensation.' Please note that this paragraph has been moved to the Methods section under the 'Study setting and population' sub-heading.*

3. **Methods**
a) 111: Clarify that it is a mixed method study design

**Response**:

*Thank you for your feedback. We have revised the sentence to better clarify the study design. It now reads, 'This was a cross-sectional observational study with a mixed-methods approach to assess the accessibility, usability, and functionality of the public HWS deployed by NBCC in 2020 in response to the COVID-19 pandemic.'*

b) 156: mention the factors relating to the functionality of the facility

**Response**:

*We have addressed this comment by adding a sentence in the methods section: 'Spot checks were conducted at all selected HWS using a structured checklist with pre-selected themes which included: location/placement, visibility and accessibility of the HWS and factors relating to the functionality of the facility such as the presence of running water, availability of soap, and functionality of the tap i.e., if it is visible and operational.'*

c) 158: Clarify how maintenance is different from sustainability

**Response**:

*Thank you for your insightful response. We modified the statement as follows: 'The caretaker survey was conducted at all HWS that had a caretaker present to evaluate the O&M of the facilities, encompassing routine tasks such as repairing broken taps or refilling soap and water to ensure immediate functionality. Additionally, the survey evaluated the sustainability of the HWS by scrutinizing broader factors like community engagement, institutional support, and resource allocation. These aspects aim to ensure the long-term viability and effectiveness of the handwashing intervention beyond the initial distribution phase.'*

d) 173 Quantitative variables is the wrong header, consider data collection and management

**Response:**

*Thank you for your suggestion. We have revised the header "Quantitative Variables" to "Data Collection and Management" to better reflect the content of the section.*

e) 183 Define status of soap, water, and tap. Also define HWSs characteristics (handwashing commodities, location, visibility, and accessibility). How was number of people seen within a HWSs measured?

**Response:**

*Thank you for your valuable feedback. We have made revisions to provide further detail on the characteristics of handwashing stations (HWSs), including handwashing commodities available, location, visibility, and accessibility.*

*Regarding the measurement of the number of people observed within HWSs, this was conducted through direct observation by trained research assistants who interviewed individuals present at each station during specific observation periods. This detailed methodology is comprehensively described in the Data Collection Methods subsection of the manuscript.*

f) 190: describe the outcome variable of the multinomial regression

**Response:**

*Thank you for your feedback. The outcome variable is clearly defined in the subsection titled 'Variables' as the effective use of handwashing stations.*

g) 198: move to section before qualitative data. How was effective use of HWS, behavioral change communication, level of knowledge and awareness of the accessibility of HWS and availability of handwashing products defined. Clarify variables by data sources- caretaker surveys, user observation, and user exit surveys.

**Response:**

*Thank you for your valuable feedback. We have repositioned the Variables subsection to precede the Quantitative Data subsection to offer a clearer understanding of the variables before delving into the explanation of both quantitative and qualitative data. Additionally, we have refined the Variables subsection to provide clarity on how the effective use of handwashing stations (HWS) was defined and how the independent variables were assessed.*

4. **Results**
a) 228-229: Confirm the distribution of the overall study population- all the exit users interviewed. The distribution of 97% by study population has limited value. Consider presenting row percentages.

**Response:**

*Thank you for your response. We have reviewed the distribution of the overall study population, specifically the exit users interviewed and modified the statement to read as follows: 'From the direct observations, the handwashing stations were used by people across different age groups, with 88.6% (365) adults observed washing their hands aged between ages 18 and 59 years, and preschools children and the elderly users were the least users, at 1% (4) and 1.9% (8) respectively.' This also incorporates the suggestion of presenting row percentages for clearer representation of the data.*

b) Table 2: Clarify what HWS accessible to children under 12yrs/ older persons means. For example, is this a height requirement?
   The footnotes do not have a reference on the table

**Response:**

*Thank you for your inquiry. The terms "HWS accessible to children under 12 years" and "HWS accessible to older persons" refer to the ease of use and reachability of handwashing stations by individuals belonging to these age groups. However, to provide clarity, we have modified the table footnote as follows: '*Accessibility was defined as HWS that are easily visible (spotted), conveniently reached by users and had visible nudges/IEC materials. HWS accessibility for children under 12 years and older persons was determined based on ease of access and suitability for use by these age groups. This designation does not imply a height requirement but rather encompasses features such as the height and accessibility of the handwashing station components (e.g., taps, soap dispensers) that facilitate ease of use for children under 12 years and older persons.' Additionally, we have made the necessary revisions to the table. The asterisks have been placed in the rightful positions corresponding to the footnotes below the table. Please note that this table is located in the supplementary file*.

c) 270-272: It is unclear how this analysis was conducted. The unit of analysis appears to be the HWS but the indicator being measured is handwashing technique. Was only one handwashing experience observed at each HWS? To clarify this discrepancy I recommend making the unit of analysis each handwashing observation (the 411 user observations).

**Response:**

*Thank you for the insightful comment. To address the discrepancy, we have revised the analysis to make the unit of analysis each handwashing observation rather than each handwashing station (HWS). It now reads, 'There was a significant difference in handwashing technique between observations made at handwashing stations (HWS) with IEC materials compared to those without (41% vs 58%, p<0.001). Each handwashing observation was treated as an individual data point for analysis.' Additionally, we have specified in the Methods section that each handwashing observation will be treated as an individual data point for analysis. The paragraph reads as follows: 'For the analysis assessing the association between the presence of information, education, and communication (IEC) materials and handwashing technique, each handwashing observation was treated as an individual data point. The unit of analysis was defined as each instance of handwashing observed at a handwashing station (HWS).'*

**Reviewer 2**:

1. **Introduction**
a) The introduction has a clear practical reason of evaluating the intervention. However, some theoretical review perhaps can be added to explain about factors that influence the effective use of technology that guide the development of study instrument and discussion.

**Response:**

*Thank you for your thoughtful feedback on our manuscript. We appreciate your suggestion to include a theoretical review in the introduction phase to explain the factors influencing the effective use of technology. After careful consideration, we believe that integrating theoretical models into the discussion section, as you suggested, would better suit the current structure of our study. While our research was not specifically based on any model, we recognize the value of incorporating theoretical frameworks to guide future investigations.*

2. **Methods**
   Very clear
3. **Results**
a) Figure 3 was very dark

**Response:**

*Thank you for your feedback. We have edited the diagram and adjusted the background color to make it more visually accessible.*

b) It is not clear which one is the results from multinomial regression model

**Response:**

*Thank you for your feedback. This has now been made clearer.*

c) Is there any analysis to look at the influence of public place and the functionality of the HWS? In some cases, more informal sites will have more risk of not functioning facilities.

**Response:**

*Thank you for your insightful recommendation. While analyzing the influence of public place on the functionality of handwashing stations (HWS) could indeed provide valuable insights, our study focused primarily on assessing the overall functionality, usability, and accessibility of HWS deployed across various settings, rather than specifically examining the differences in functionality based on the type of public place. However, for future research, exploring the influence of public place on HWS functionality could be a valuable avenue for investigation.*

4. **Discussion**
a) It has provide good discussion about the HWS provision intervention and how to increase lifetime and usability. It would be better to integrate the discussion with any model or theory to discuss the importance of all significant factors. Appropriate technology criteria, sustainable technology concept, or technology acceptance model can be some of important concept can be used in discussing the findings.

**Response:**

*Thank you for your insightful suggestion. As stated in our earlier response, we acknowledge the importance of integrating the discussion with existing models or theories such as the technology acceptance model to contextualize our findings and understanding their implications. We have incorporated this relevant model into our discussion to provide a more comprehensive analysis of the significance of the identified factors and their implications for future interventions. This modification is located in the last paragraph of the discussion section.*

5. **Conclusion**
a) Would be better to have a sub-title for the conclusion

**Response:**

*Thank you for your feedback. We have revised the manuscript accordingly. The last paragraph in the discussion section now serves as the conclusion section, comprising the key findings and implications of the study.*
